# Fast ^1^H-NMR Species Differentiation Method for *Camellia* Seed Oils Applied to Spanish Ornamentals Plants. Comparison with Traditional Gas Chromatography

**DOI:** 10.3390/plants10101984

**Published:** 2021-09-23

**Authors:** Rocío Barreiro, Raquel Rodríguez-Solana, Leocadio Alonso, Carmen Salinero, José Ignacio López Sánchez, Efrén Pérez-Santín

**Affiliations:** 1 Estación Fitopatolóxica Areeiro, Deputación de Pontevedra, Subida á Carballeira, 36153 Pontevedra, Spain; robarreiro@uvigo.es (R.B.); carmen.salinero@depo.es (C.S.); 2 Departamento de Química Orgánica, CINBIO, Universidade de Vigo, Campus Universitario As Lagoas, Marcosende, 36310 Vigo, Spain; 3 Department of Food Science and Health, Andalusian Institute of Agricultural and Fisheries Research and Training (IFAPA), Avda. Menéndez Pidal s/n, 14004 Cordoba, Spain; raquel.rodriguez.solana@juntadeandalucia.es or; 4 Faculdade de Ciências e Tecnologia, MED–Mediterranean Institute for Agriculture, Environment and Development, Universidade do Algarve, 8005-139 Faro, Portugal; 5 Instituto de Productos Lácteos de Asturias (CSIC), Paseo Río Linares s/n, 33300 Villaviciosa, Spain; lalonso@ipla.csic.es; 6 Escuela Superior de Ingeniería y Tecnología, Universidad Internacional de La Rioja, Avda. de la Paz, 137, 26004 Logrono, Spain; joseignacio.lopez@unir.net

**Keywords:** *Camellia* oil, authentication, quality, chromatographic techniques, nuclear magnetic resonance, chemometrics

## Abstract

*Camellia* genus (Theaceae) is comprised of world famous ornamental flowering plants. *C. japonica* L. and *C. sasanqua* Thunb are the most cultivated species due to their good adaptation. The commercial interest in this plant linked to its seed oil increased in the last few years due to its health attributes, which significantly depend on different aspects such as species and environmental conditions. Therefore, it is essential to develop fast and reliable methods to distinguish between different varieties and ensure the quality of *Camellia* seed oils. The present work explores the study of *Camellia* seed oils by species and location. Two standardized gas chromatography methods were applied and compared with that of data obtained from proton nuclear magnetic resonance spectroscopy (^1^H-NMR) for fatty acids profiling. The principal component analysis indicated that the proposed ^1^H-NMR methodology can be quickly and reliably applied to separate specific *Camellia* species, which could be extended to other species in future works.

## 1. Introduction

*Camellia* is a genus of flowering plants in the family *Theaceae*, native to East Asia and widely distributed in China, India, Japan, and South-East Asian countries, whose seeds and leaves present high nutritional and medicinal values. This subtropical evergreen shrub or small tree arrived in Europe around the 16th century [1], and was introduced into the gardens of the highest social classes of Galicia (NW of Spain) at the beginning of the 19th.

Nowadays, cultivars of *Camellia* species are found worldwide in public and private gardens thanks to their excellent adaptation to climatic and edaphic conditions, easy spread, and resistance to pests and diseases. Particularly, *Camellia japonica* L. is the best known internationally as a cultivated species for ornamental value. In the last decade, commercial interest was remarkable, and consequently, production in Spain reached about 2.5 million *Camellia* plants per year, which are exported throughout Europe as ornamentals [2,3,4].

*Camellia* oil is obtained from the seeds, known as one of the most popular edible vegetable oils that was utilized for more than 1000 years in China, and also abundantly used in southeast Asian countries (Japan, Korea, India, Sri Lanka, Indonesia, and Vietnam), where *Camellias* are abundantly available [5].

*Camellia* oil is also known as “Eastern Olive Oil” because it shares a similar chemical composition with olive oil [6]. It contains several natural antioxidants, such as squalene, phytosterol, polyphenols, fat-soluble vitamins (vitamins A, B, E), sasanqua saponin, and other functional substances. It was recommended by the Food and Agriculture Organization of the United Nations as a high-quality, healthy vegetable oil because of its nutritional value and excellent storage qualities [7]. For these reasons, it is commonly used as cooking oil (edible oil) [8,9]. In China, the main species used for oil production is *Camellia oleifera* C. Abel [10], while in Japan this is *C. japonica* [11], and *C. sasanqua* in Vietnam [12].

*Camellia* oil is an expensive product with a particular and characteristic aroma and taste, good storage stability, and high nutritional and medicinal values, with high value interest for trade [13]. Thus, the economic interest in this crop increased exponentially in recent years for a variety of purposes [14]. Specifically, *Camellia* oil extracted from seeds of different species, including *C. reticulata Lindl.*, *C. sinensis* L., *C. oleifera*, and *C. japonica*, was long processed as an industrial oil used for oligosaccharide production [15], as a surfactant, in soaps, as a hair oil, and now it is generating interest as a biofuel source, lubricant, and biopolymer [16,17,18,19,20]. Although, in cosmetics *C. japonica* oil has a long history of traditional cosmetic usage in Japan as a protectant to maintain skin and hair health, where other species are nowadays commonly used for this purpose (e.g., *C. oleifera, C. grijsii* Hance, and *C. sasanqua*) [11,21]. *Camellia* oil has fat-soluble natural compounds with health benefits, reducing cholesterol and triglycerides in the blood, lowering blood pressure, and promoting effects such as antioxidation, antipermeability, anti-inflammation, as an analgesic, and anticancer properties [22,23,24], as well as antimicrobial and antiviral activities [25]. In addition to this, they are used in traditional treatments in China to prevent cardiovascular diseases, arteriosclerosis, and burn injuries [26,27,28].

Triacylglycerols are the principal components of *Camellia* oils, with a high proportion of oleic and linoleic acids and low saturated acids. This general lipidic profile is associated with well-known health properties. The oil yield of seeds from this plant is high, being on average 30% oil per seed. However, the seed oil content varies according to species, cultivar, and environmental conditions [29,30]. The profile of fatty acids (FAs) allows correlation to be made with their botanical origin, which is a very important aspect from a commercial point of view, since the traceability of these oils is mandatory to avoid fraud by adulteration. The properties of the oils are also dependent on the FAs’ composition. The degree of unsaturation and chain length, and the presence of polyunsaturated FAs, appear to increase the potential beneficial properties of these oils [31]. The unsaturated FAs content in *Camellia* oil can reach as much as 90%, which is the highest amount so far reported for unsaturated FAs in edible oils [22,32,33]. In recent years, *Camellia* oil became one of the most popular and expensive edible vegetable oils on the market in China, being more susceptible to adulteration with other cheaper oils by unscrupulous traders for high profits. Another aspect of fraud, the mislabeling of oil extraction methods, and geographical or origin, also destabilize the local *Camellia* oil market economies [34]. The method for *Camellia* oil authentication currently used officially, employing gas chromatography (GC) techniques, includes the FAs’ composition. The increased demand for *Camellia* oil made the development of rapid and reliable methods for the unequivocal chemical plant species oil characterization associated with the quality of the edible oil a priority objective to avoid commercialization of adulterated *Camellia* oils [35,36,37,38,39].

To determine the FA composition, a wide variety of analytical methods are available. In this context, traditional methods are gas chromatography with flame ionization detectors (GC-FID) [40] or gas chromatography-mass spectrometry (GC-MS) [41]. In these methods, a pretreatment of the sample is necessary to convert the FA into the corresponding methyl esters (FAMEs). So, these methodologies are tedious, time-consuming, require the use of FAs standards, and involve complicated pretreatment of the samples prior to analysis, such as the triacylglycerol hydrolysis and esterification that could face problems of oxidation during the derivatization process [42,43,44].

Currently, new, rapid, and nondestructive methods such as Near-InfraRed (NIR), Raman Spectroscopy, and Nuclear Magnetic Resonance (NMR) techniques were recognized as alternative analytical tools in combination with appropriate chemometrics in oil quality control [45]. Specifically, recent studies confirmed that NMR is a powerful tool for qualitative and quantitative analysis of FAs composition in edible vegetable oils [32,40,46,47,48,49,50].

Therefore, the aim of this study was to compare different analytical techniques, including chemical (quality parameters), chromatographic, and nuclear magnetic resonance methods, for the study of several species of *Camellia* seed oils harvested in Spain. The geographical traceability and species origin of *Camellia* oil was corroborated. Finally, the suitability of each of the analytical techniques applied in relation to its species grouping of oils according to their chemical profile was evaluated through the principal component analysis.

## 2. Results and Discussion

### 2.1. Oil Content

Seeds of all *Camellia* species contain oil. However, oil content and quality may vary with species [51]. High seed oil variability is likely the result of several factors, including environmental variables such as soil, altitude, light, rainfall, humidity, and temperature, all playing a key role, as previously demonstrated for a variety of plants [30]. Thus, seed oil content (SOC) of traditional *Camellia* varieties can range between 24% and 50%, with an average about 30% [29]. *C. oleifera,* which is the earliest species exploited for edible oil, accounting for 98% of the *Camellia* cultivated area in China, was previously reported to provide an SOC between 21% and 34% [52]. Moreover, some of the new *C. oleifera* cultivars can reach as much as 53% oil per dry seed [53].

**Figure 1:**
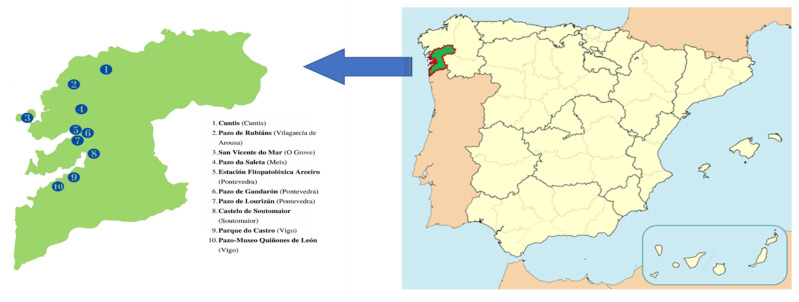
*Camellia* locations.

In this study, seeds from different *Camellia* species (*C. japonica*, *C. sasanqua*, *C. reticulata*, and *C. hiemalis* Nakai) were harvested in various locations in the province of Pontevedra (Galicia, NW Spain, Figure 1) during the last four months of 2019. The percentage of seed oil extracted from *Camellias* varied from 16.1% to 31.9% for *C. japonica*, and from 22% to 30.1% for *C. sasanqua*, providing mean values of 23.1% and 25.8%, respectively (Table 1). Thus, both species are appropriate candidates for use in *Camellia* oil production. *C. reticulata* and *C. hiemalis* showed slightly lower values of 16.6% and 22.6%, respectively.

### 2.2. Quality Index Parameters

The quality of *Camellia* oil is greatly influenced by extraction technologies [54]. Cold-pressing is generally one of the most common traditional methods to produce healthy *Camellia* oil [51]. Acid value is an important index of the quality of edible oils, providing information about the free FAs content in lipids. Usually, the lowest acid value is related to the best oil quality and oxidation stability, while high values due to free FAs lead to decreased thermal and oxidative stability. Even though *Camellia* oil is not currently regulated at the European level as an edible oil, this parameter was determined for all *Camellia* oils in this study to compare with the standard values legislated by the official olive oil method, according to the Spanish and International regulation [55]. Thus, Extra Virgin Olive Oil must have an acid value lower than 6.0 mg KOH/g oil. Table 1 shows mean acid values obtained for each of the camelia species studied, ranging from 0.39–5.66 mg KOH/g oil. Thus, *Camellia* oils showed low values, below the maximum authorized in olive oil for food/industrial purposes. Among species, *C. japonica*, with a greater number of samples analyzed, presented great variability in its composition (Table 1), with the oils from EFA being the ones that presented the lowest values (0.39 and 1.81 mg KOH/g oil). These results were also similar to the one (1.7 mg/g) found in the literature for the same species [56].

Iodine value is also an oil quality index representative of the number of unsaturated C-C bonds from FAs. Results obtained for the iodine index of *Camellia* oils were compared with those set by the official method for olive oil, ranging from 70.3 to 92.0 g I_2_/100 g oil (Table 1). There is no regulation for *Camellia* oil in Spain, but values between 75 and 90 g I_2_/100 g oil are set as healthy by Spanish legislation, and therefore they were used as a reference [55]. Thus, iodine values obtained for the different species of *Camellia* oils were, in general, similar to those referred to as healthy by Spanish legislation, with only two samples (S12 and S16) out of this range, since they showed iodine values slightly out of this range (Sample 12, Pazo de Rubiáns–Bento de Amorim, with 70.3 ± 0.4, and Sample 16, *C. sasanqua* from Pazo de A Saleta, with a value of 92.0 ± 0.5). Furthermore, the values obtained in *C. japonica* were really close to that of 79.9 g/100 g obtained by Zeng and Endo, (2019) [56] for the same species.

### 2.3. GC-FID Analysis

FAs composition is one of the most important indexes in edible oils, closely related to their price [57]. The proportion of saturated and unsaturated FAs varies in edible oils. This FAs profile of edible oils is closely related to lipid oxidation, product quality, and function of vegetable oils. Thus, highly unsaturated FAs’ (UFAs) oil content is more expensive because consumers assume that they are healthier. Furthermore, the price of edible oils is different in any place depending on factors such as the local availability of the vegetable source needed to extract the oils, the mechanization of agriculture, and the economy of the oil production area, among others [51]. For example, the price of olive oil with a fairly mechanized production and cultivated in large areas of the south of Europe is relatively higher than that of soybean oil produced mainly in China, US, Argentina, and Brazil, with the latter more expensive than palm oil, which is the most widely consumed vegetable oil. Indonesia and Malaysia are the top palm oil producers, followed by Thailand, Nigeria, and Colombia.

*Camellia* oil has a very similar FAs profile and physicochemical properties to olive oil, being given with the designation of “oriental olive oil”. It is rich in UFAs (>90%), especially oleic acid (74–87%), as well as in other type of compounds such as polyphenols, fat-soluble vitamins (Vitamins A, B, E), and minor unsaponifiable matters (2–5%), including squalene and phytosterol, etc., [51,58].

In this work, the FAs composition of *Camellia* oils from different species were analyzed by GC-FID as methylated derivatives (FAMEs) and the results expressed as mean values ± standard deviations as shown in Table 2. All tested samples contained similar FAs composition, showing nine common FAs compounds. Among them, oleic (C18:1), palmitic (C16:0), linoleic (C18:2), and stearic (C18:0) acids were the predominant FAs, which accounted for 98.5–99.5% of the total, similarly to the results found for total FAs composition of extra virgin olive oil (97.5%) used as a control. Oleic acid (C18:1) was the major component in *Camellia* samples, ranging from 77.9% to 83.6%, followed by palmitic acid (C16:0, 8.2% to 10.8%), linoleic acid (C18:2, 3.9% to 8.0%), stearic acid (C18:0, 1.7% to 3.9%), and linolenic acid (C18:3, 0.23% to 0.45%). Other fatty acids, such as myristic (C14:0), palmitoleic (C16:1), arachidic (20:0), and eicosenoic (C20:1) acids, were found in concentrations lower than 0.2%. Due to the *Camellia* oil characteristics based on a high oleic acid content and the presence of essential fatty acids (C18:2 and C18:3), which cannot be synthesized by the human body and need to be solely supplied through diet, *Camellia* oils may provide health functions, such as the lowering of blood pressure, cholesterol, and triglycerides, and thus prevent cardiovascular diseases, cancer, hypertension, and autoimmune disorders. It is also of value in protecting the liver against peroxidative damage, as was stated by the carbon tetrachloride-induced hepatotoxicity model [59].

According to the species used in oil production in China, it was found that the composition of *C. japonica* was rich in oleic acid (C18:1) with values of 86.6%, followed by palmitic acid (C16:0; 7.5%), linoleic acid (C18:2; 3.0%), and stearic acid (C18:0, 2.1%), and showed low quantities of palmitoleic acid (C16:1), linolenic acid (C18:3), and arachidic acid (C20:0) in all of them with a proportion of 0.1%, and erucic acid (C22:1) (0.3%) [56]. In reference to our results, the *C. japonica* samples showed a slight decrease in the content of oleic acid and an increase in palmitic acid, as well as a higher concentration of essential fatty acids, namely linoleic acid (C18:2) and linolenic acid (C18:3). The oleic acid values found in *C. japonica* were higher than in that of other species of *Camellia,* such as *C. oleifera* and *C. sinensis,* with values of 80.5 and 58.4%, respectively, and even the oleic acid in olive oils, which showed values between 54.1 and 75.5% [60].

Also, slight differences between total saturated fatty acids (SFA), monounsaturated fatty acids (MUFA), and polyunsaturated fatty acids (PUFA) were found. All *Camellia* oils showed low values of SFA (10.2–13.6%), mainly for palmitic acid (C16:0) (Table 2). The SFA in *C. Japonica* was in the range of 7.3% to 9.5%, while *C. Sasanqua* showed higher values between 10.8% and 12.8%. *C. reticulata* and *C. hiemalis* presented SFA values of 13.6% and 12.1%, respectively. The MUFA content is mainly due to the contribution of oleic acid, with a minor contribution from other monounsaturated acids, with *C. japonica* being the species with the highest percentage in reference to the other species studied, 79.3% to 84.2% and 78.4% to 81.2%, respectively. However, this trend is the opposite in the case of PUFA, showing values from 4.2% to 7.7% in *C. Japonica*, while the values were higher in the other species, ranging between 7.1% and 8.3%. In general, oleic acid (C18:1) is usually considered to be more stable than linoleic (C18:2) and linolenic acid (C18:3). The results showed that *Camellia* oils contained high levels of MUFA and low PUFA, favoring the nonappearance of unpleasant odors due to oxidation. Therefore, this may be a justification of the suitability of this oils for cosmetic applications and for cooking at high temperatures [56].

### 2.4. GC-MS Analysis

Gas chromatography-mass spectrometry is a practical and powerful analytical technique used for the quantification of fatty acids, and also commonly used as a separating criterion for *Camellia* oil authentication [61]. The results obtained using the method based on GC-MS (Table 3) were analogous to those using the GC-FID methodology previously described. However, some differences were found. Although the values for the main compounds, namely oleic (C18:1), palmitic (C16:0), linoleic (C18:2), and stearic (C18:0) acids showed similar ranges in both techniques, the minor fatty acids myristic (C14:0), palmitic (C16:1), linolenic (C18:3), and arachidic (C20:0) acids presented values lower than 0.2%, and therefore, they were not quantified. The limits of quantification from GC-MS are usually higher than those from GC-FID. For example, Dodds et al., (2005) [62] found for standard FAMES that the limit of quantification (LOQ) of myristic acid (C14:0) is five times higher for GC-MS than that of GC-FID, e.g., 2.52 pmol and 0.50 pmol, respectively. Also, higher LOQs were found by GC-MS for the compounds palmitic (C16:1), linolenic (C18:3), and arachidic (C20:0) acids, which, due to the low concentrations found in the samples, did not allow for their quantification.

However, the quantification of FAMEs by GC-MS offers two powerful advantages over GC-FID, namely the ability to confirm the identity of analytes based on spectral information, retention time, and the ability to separate peaks from a noisy background, or coeluting peaks if unique ions are available [62]. The results indicate that GC with a mass detector allowed for the identification and quantification of two positional isomers of oleic fatty acid (C18:1 ω-9 *cis* and *trans*) due to its different fragmentation profiles, while with GC-FID this was not possible.

The oils found in nature are in the form of triglycerides, fatty acids generally found with saturated and unsaturated bonds, and the FAs containing double bonds are usually stable as cis isomers. A small percentage of these acids can isomerize to their *trans* configuration during the extraction, refinement, or hydrogenation processes. The *cis* configuration is nutritionally important, while the conversion into *trans* from *cis* is reported to have adverse effects on human serum lipoproteins and contributes to increasing the risk of coronary heart disease [63]. Our results showed very low amounts of C18:1 ω-9 *trans* (from 0.42% to 1.18% depending on the species) in all samples. In contrast, the presence of C18:1 ω-9 *cis* was higher, with values ranging between 83.3% and 89.2%. This is of great importance due to the different healthy properties of this compound found in high quantities in *Camellia* oils.

MS-chromatographic techniques were widely employed in oil quality and safety assessments, with a high specificity and sensitivity to quantify those targeted analytes (FAs) to have a rigorous control (authentication and classification) of samples. However, as in the case of the GC-FID technique, it involves tedious, destructive, and extensive sample preparation. So, these conventional chromatographic techniques have a number of limitations for further quality control oil applications.

### 2.5. H-NMR Analysis

The NMR spectroscopy was extensively used for oil analysis, and it was established as a valuable tool for the assessment of the quality and authenticity of olive oil [64,65]. NMR was used to develop accurate analytical fingerprinting methods for the authentication or certification of the geographical origin of olive oils aided by suitable chemometric analysis [66,67]. Studies of time, thermal, and oxidative stability of olive oils by NMR analysis were also powered by multiway chemometric methodologies [68,69]. Also, ^1^H-NMR combined with chemometrics were employed for the prediction of fatty acid composition [50], to detect the adulteration of *Camellia* oil [49], and to determine oxidative stability in *Camellia* oils [70].

In previous work, Feás et al., (2013) [32] determined the FA profile of three species of Galician *Camellia* oils (*C. oleifera, C. reticulata* and *C. sasanqua*, see Table 4 samples 21–23) collected at the *Estación Fitopatolóxica do Areeiro* in 2011, with values ranging between 82.3% and 84.5%, 5.69% and 7.78%, 0.26% and 0.41%, and 8.04% and 11.2%, for oleic, linoleic, linolenic, and saturated acids, respectively. These values demonstrate that the FAs composition remained fairly stable over time for these species in the region. In this methodology, Feás et al. used the tertiary hydrogen of the glyceryl group (δ 5.25 ppm) as the key indicator to estimate the FAs composition. The magnetic field for providing good results was established as 17.6 T (750 MHz) to avoid signal overlapping of protons of the acyl and glyceryl groups (5.32 and 5.25 ppm, respectively, see Table 5). However, the NMR equipment at 750 MHz is of high cost, which would make the technique not easily available and therefore not applicable. To improve the applicability of the ^1^H-NMR technique for the determination of the FA composition in *Camellia* oils, an adaptation of the Barison method was carried out in the present work taking as reference a more common NMR instrument of 400 MHz [71] (Table 6).

Fatty acid compositions found in *Camellia* oils are shown in Table 4. *Camellia* oil samples showed values ranging from 81.0% to 98.1%, 4.33% to 10.4%, and 11.6% to 17.3% for oleic acid (C18:1), linoleic acid (C18:2), and saturated acids, respectively. In most cases, the fatty acid contents found were close to the levels showed in chromatographic analysis and comparable with data from the literature based on NMR analysis of Galician *Camellia* oils [3,32]. In general, the content of oleic acid (C18:1) in *C. Japonica* (91.4%) and *C. hiemalis* (91.1%) showed average values higher than in *C. sasanqua* (84.5%) and *C. reticulata* (81.0%), although *C. japonica* showed a wide variability, including that of linoleic acid in the range 4.3–7.3%. No significant amounts of linolenic acid (C18:3) were detected. The slight differences in the FA profile between chromatographic and NMR samples may be due to the approximations implied in Barison’s method based on two approaches: (1) All fatty acid acyl chains were esterified on the glycerol moiety, and (2) there were no free fatty acids in the samples [71]. In relation to this, neither di- nor monoacylglycerols were detected, as confirmed by the absence of peaks in the spectrum at 4.12 and 2.27 ppm, respectively. Also, the acid value in all *Camellia* oil samples is lower than 6 mg KOH/g of oil, and therefore *Camellia* oils are optimal candidates for the application of this methodology.

**Figure 2:**
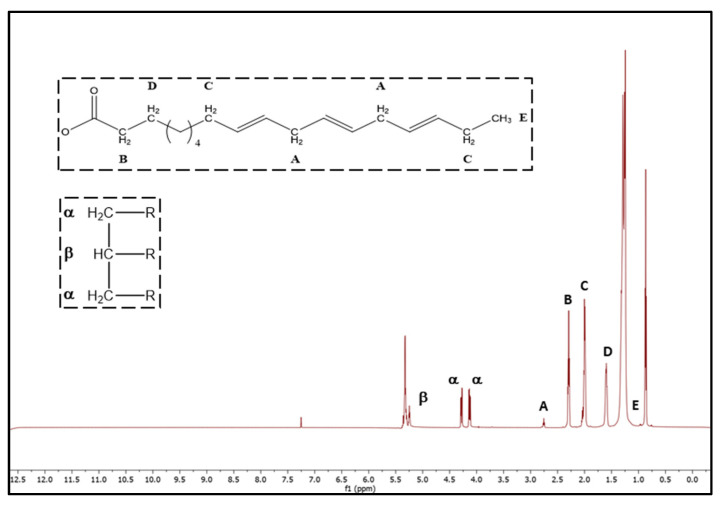
Structure of FAs and ^1^H-NMR spectrum at 400 MHz of camelia seed oil.

The application of the ^1^H-NMR methodology developed to determine FA content in *Camellia* oils is simpler and faster than conventional methods due to the absence of sample pretreatment, low-reagent consumption, short analysis (approx. 3–4 min), excellent repeatability, and fully automatic routine protocol in the NMR software [20,50,70]. Although currently the costs per sample are affordable, however, professional operating personnel are necessary. Moreover, this technique avoids problems such as lipid oxidation present in the traditional GC analysis, it does not require the use of standards, it is a nondestructive technique, and it provides information about distribution of FAs (Figure 2) [72,73,74].

### 2.6. Principal Component Analysis (PCA)

**Figure 3:**
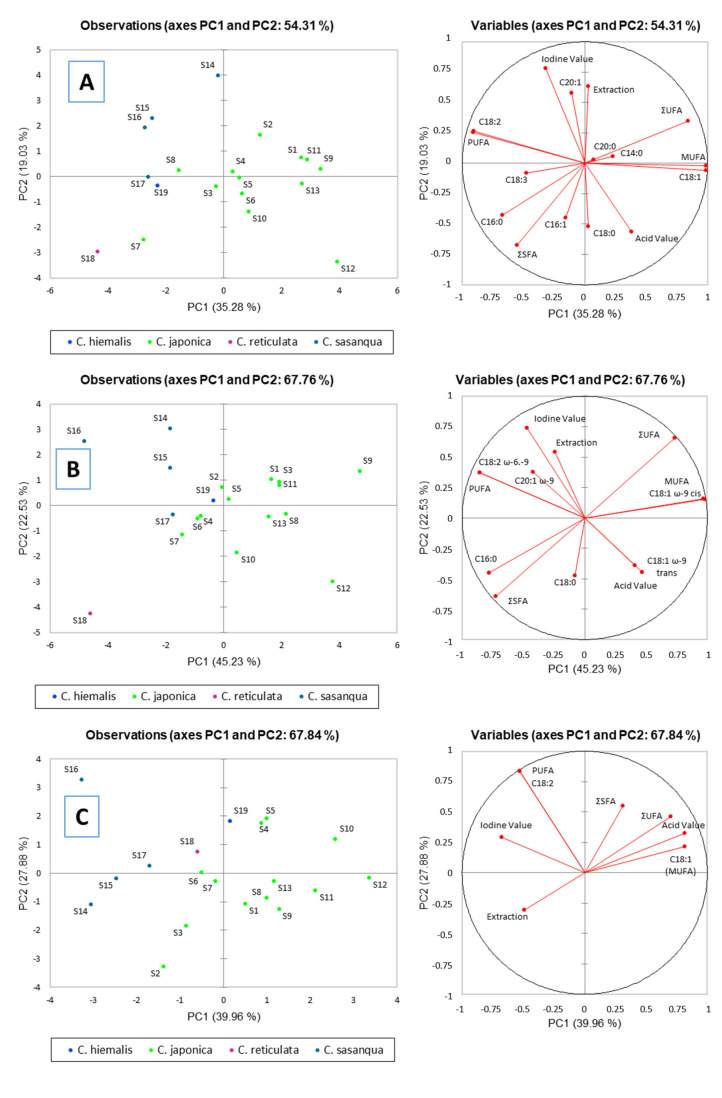
Principal component analysis plot of *Camellia* oils from different species. FAs were determined by (**A**) GC-FID, (**B**) GC-MS, (**C**) ^1^H-NMR.

Principal component analysis (PCA) was used to identify the parameters, mainly fatty acids, that better separate 19 seed oils from four species of *Camellia*, namely the most widespread *C. japonica* and *C. sasanqua*, and the less common species *C. reticulata* and *C. hiemalis*. Figure 3A–C show the biplot of the two main principal components (PC1 and PC2) characterized by the common parameters studied in samples including iodine and acid values, extraction efficiency, and the FAs profile studied with the gas chromatography techniques (GC-FID and GC-MS) and the proton nuclear magnetic resonance technique (^1^H-NMR). This FA profile presented saturated FAs (C14:0, C16:0, C18:0 and C20:0), total saturated FA (∑SFA), total unsaturated FAs (C16:1, C18:1, C18:2, C18:3, and C20:1), total monounsaturated FA (MUFA), total polyunsaturated FA (PUFA), and total unsaturated FA (∑UFA). The cumulative explained total variance ranged from 54.31% (GC-FID) and 67.76% (GC-MS) for the chromatographic techniques to 67.84% for ^1^H-NMR technique.

#### 2.6.1. Chromatographic Techniques

Figure 3A,B correspond to the PCA obtained with data from GC-FID and GC-MS techniques, respectively. In general, the observed trend found between the two techniques is similar in terms of differentiation of species according to the mentioned parameters evaluated in samples, those being seed oils from *C. japonica* with the highest values in unsaturated FAs and acid contents. According to the GC-FID and GC-MS data, samples corresponding to the *C. japonica* species are distributed in both principal components (PC1 and PC2), quadrants I–IV. This species, despite being dispersed throughout practically the entire PCA, differs perfectly from the rest of the species studied, namely *C. reticulata*, *C. hiemalis*, and *C. sasanqua*. In general, in both chromatographic techniques, oils from 1, 9, 11–13 *C. japonica* samples are defined by the high content in the unsaturated C18:1 and total ∑UFA and MUFA contents. Sample 10 and 12 are mostly defined by high-acidity and saturated C18:0 FA contents, while sample 7 is better characterized by its highly saturated FA content (the C16:0, and total ∑SFA contents). According to GC-FID results, samples 4–6 and 10 are characterized by their highly saturated C14:0 and C20:0 FA contents. In relation to *C. sasanqua* species, these oils presented the highest iodine and extraction yields, and contents in unsaturated FAs (C18:2, C20:1, and total PUFA). *C. reticulata* oil (sample 18) stands out for its C16:0 and ∑SFA contents (in the same way as in sample 7 from *C. japonica* species). Finally, as can be observed for the position of sample 19 that belongs to *C. hiemalis,* this oil presents an intermediary profile between those of *C. sasanqua* and *C. japonica* species. According to GC-FID data, this oil is more characteristic for its C18:3 content and presented a profile similar to that of samples 17 (*C. sasanqua*) and 8 (*C. japonica*). However, according to PCA with GC-MS data, this sample presented a profile similar to those of different *C. japonica* samples (2, 4–6).

#### 2.6.2. Proton Nuclear Magnetic Resonance Technique

Similar to the distribution observed in chromatographic techniques, ^1^H-NMR showed in general a clear distinction among *Camellia* seed oils obtained from different species. For this technique, in comparison with that of chromatographic techniques, the obtained grouping of sample oils according to their species origin is achieved simply with 6 characterized FAs, compared to the 12 FAs of GC-MS and 13 from GC-FID.

In the case of oils from *C. japonica* seeds, again they were distributed in all quadrants (I–IV). Among these samples, 4–5 and 10–12 are mainly characterized by their high C18:1 (MUFA) and acid values, as well as their total saturated and unsaturated (∑SFA and ∑UFA) FAs contents. According to *C. sasanqua* oils, these samples could be defined by their highest PUFA and C18:2 values (more specifically sample 16), and high iodine and extraction yield (highlighting sample 14) values. On the other hand, the sample from *C. hiemalis* (sample 19) presented a chemical profile more characteristic from *C. japonica* samples (samples 4 and 5). *C. reticulata* (sample 18) presented similarities in the FA profile (C18:2 and PUFA) and iodine value with samples 17 and 6 corresponding to *C. sasanqua* and *C. japonica* oils, respectively.

With this technique, the similar composition of samples from the same geographical location is more evident, and therefore, samples from Pazo de Rubiáns (samples 9–13) are proximal in the graph (quadrants I and IV of the PCA). In the same way, oils confectioned with plants from other sampling locations [EFA (samples 2 and 3 from *C. japonica*, and 14 and 15 from *C. sasanqua*) and Pazo Quiñones de León (samples 4 and 5 from *C. japonica*)] are also close in the graph.

## 3. Materials and Methods

### 3.1. Materials and Reagents

Silica gel for column chromatography (0.063–0.22 mm), activated carbon (100 mesh particle size, powder), deuterated chloroform (CDCl_3_), and FAME Mix (C14-C22) certified reference material were purchased from Merck (Madrid, Spain). Wijs (iodine monochloride) solution (ICl, 0.1 M) was purchased from Scharlab (Barcelona, Spain). Deionized water was obtained in the laboratory by using the Millipore Q3 Ultrapure Water Distiller (Merck, Darmstadt, Germany). Reference material CRM-162 was obtained from the European Commission, Brussels, Belgium.

### 3.2. Plant Material

The selection was based on the abundance and availability of each species in the Galician landscape, where *C. japonica* and *C. sasanqua* are the major species used as ornamental plants, while the other species, such as *C. reticulata* and *C. hiemalis*, are less interesting because they present less availability and low seed oil in comparison with that of the aforementioned species [5,32,75]. Samples from *Estación Fitopatolóxica Areeiro* (EFA) of these species were harvested in different zones labelled 826 and 942. The harvesting was carried out when fruits began to split open and the seeds were visible, a phenological stage of fruit development that corresponds to the *Biologische Bundesantalt* and *Chemische* (BBCH scale) [2]. Sampling was carried out in a stratified random fashion within the populations. More than 400 plants were sampled for the study. From each individual sample plant, at least 30 mature fruits were randomly selected for further analysis.

### 3.3. Camellia Seed Oil

The crude *Camellia* seed oil was extracted by the traditional mechanical pressing method that is still widely adopted for the commercial processing of *Camellia* seed oil. *Camellia* seeds were washed in water and dried at 22 °C for 48 h followed by the mechanical crushing process. Oil extraction was performed using a mild, cold-pressed method. Approximately, 2.5 kg of dried *Camellia* seeds were transferred to the automatic hydraulic press (Honmac 6YZ-260, Zhenfzhou City, China), and then pressed to 55 MPa for 5 min to obtain the oil. Subsequently, oils were filtered through cellulose, silica gel, and an activated carbon filter under a vacuum (*p* < 2 mbar) using a vacuum pump (ILMAC FB65454, Fisher Scientific, Madrid, Spain) to remove oil impurities and then were stored in amber bottles at room temperature. The oil samples were weighed, and the yield was expressed as mass of extracted oil per mass of dried seed in a percentage.

### 3.4. Determination of Acid Value

The acid value (AV) was determined according to the standard method ISO 660:2009 [76]. The method is based on the titration of a solution of 10 g of *Camellia* oil dissolved in ethanol/diethyl ether (1:1, *v*/*v*) with a KOH solution (0.1 M in ethanol), using phenolphthalein as indicator. Results were expressed as mg of KOH per 1 g of oil. All determinations were carried out in triplicate.

### 3.5. Determination of Iodine Value

The iodine value (IV) was calculated according to the standard method ISO 3961:2018 [77]. About 0.20 g of sample oil was dissolved in a mixture of cyclohexane and glacial acetic acid (50:50, *v*/*v*). Then, 25 mL of Wijs solution were added and the mixture was maintained during 1 h in the dark. Finally, the excess of iodine generated was titrated with sodium thiosulfate with the previous addition of 20 mL of potassium iodide and 150 mL of deionized water. Results were expressed as grams of iodine per 100 g of oil. All determinations were carried out in triplicate.

### 3.6. FAMEs Preparation and Analysis by GC-FID

The preparation and analysis of FA methyl esters (FAMEs) were based on the method proposed by Alonso et al., (2000) [78]. About 100 mg of *Camellia* oil was weighed and dissolved in 1 ml of hexane. Then, 0.1 ml of methanolic potassium hydroxide (2 M) was added and the mixture was stirred for 1 min and left to rest for 15 min. Next, the hexane layer was separated, and 0.1μL of the hexane fraction was injected into the GC.

The GC analysis of FAME was performed on an Agilent Technologies GC Agilent Technology 5975 B (Palo Alto, CA, USA) equipped with a flame-ionization detector (FID). Analyses were performed with a CP Sil 88 column (100 m × 0.25 mm i.d.) containing 100% cyanopropyl siloxane, stationary phase, with 0.20μm film thickness (Chrompack, Middelburg, The Netherlands). The initial temperature of 175 °C was maintained for 28 min, then raised to 210 °C at a rate of 1.3 °C/min for 10 min. The split ratio was 1:50, and the carrier gas was helium with a flow rate of 1 ml/min. The injector and detector temperatures were 250 °C. For quantitative determinations of total FAMEs, anhydrous soy, corn oil blend with a certified FAs composition (reference material CRM-162) was used. All determinations were performed in duplicate.

### 3.7. FAMEs Preparation and Analysis by GC-MS

FAMEs were prepared according to International Olive Council IOC/T.20/Doc 24 protocol [79], with some modifications. A solution of 0.1 g of the sample oil in 2 ml of heptane was vortexed for 1 min. Then 0.2 ml of methanolic potassium hydroxide solution (2 M) was added. The solution was vortexed vigorously for 30 s. When the solution was stratified, the upper layer with methyl esters was separated. An aliquot was filtered through a 0.45μm Polyvinylidene Fluoride (PVDF) filter. The analyses were performed in triplicate.

According to the International Olive Oil Council COI/T.20/Doc. 33 protocol [80], FAMEs were separated and quantified using an Agilent GC-7890B coupled to MSD-5977A detector instrument (Agilent Technologies, Santa Clara, CA, USA) with an HP-5MS (5%-phenyl) methylpolysiloxane, length 30 m × 0.25 mm i.d. (0.25 μm film thickness) capillary column (Agilent Technologies, Santa Clara, CA, USA). The analysis conditions were as follows: the temperature of the injector was set at 250 °C, and the injection volume was 1 μL with 1:100 split ratio. Helium was used as the carrier gas at a flow rate of 1 ml/min. A temperature program with injection at 165 °C was held for 8 min, and then raised at a gradient of 2 °C/min to 280 °C with 2 min hold, with 37 min total run time. The electron ionization source was 70 eV in negative voltage at 230 °C, and the range of the mass detector was set from 40 up to 850 *m/z*. They were quantified according to their percentage area, obtained by the integration of the peaks. The results were expressed as the percentages of individual FAs. FAMEs were identified through a comparison of retention times of pure standards analyzed under the same conditions. MassHunter Software Version B.07.00 was used to control and process the obtained data. The identification of compounds was achieved by comparing the retention index with the spectral data obtained from Mass Spectral Library Version 2.0 g (NIST-MS, 2012, Agilent Technologies, Santa Clara, CA, USA).

### 3.8. FA Analysis by ^1^H-NMR

The ^1^H-NMR analysis was carried out according to the method described by Barison et al., (2010) [71]. The determinations of FAs composition by ^1^H-NMR spectroscopy (Figure 2) were performed on a Bruker AVANCE ARX400 NMR spectrometer operating at 9.4 T observing the ^1^H nuclei at 400 MHz. About 200 μL of each oil sample was directly transferred into 5 mm OD Lab Class Precision NMR sample tube (Wilmad Labglass Inc. USA), and the volume was completed to 600 μL with CDCl_3_ and shaken in a vortex mixer for 30 s. The temperature of the sample in the probe was maintained at 30 °C. The relaxation delay (14 s) and pulse (20°) were parameters equally fixed in both instruments. The chemical shifts are reported in ppm, calibrated by setting the peak of tetramethylsilane as an internal reference (δ = 0.00 ppm). Phase and baseline corrections were performed automatically to ensure a better quantitative comparison of the spectra. The spectra were integrated by Mestrenova software (ver. 12, Mestrelab Research SL, Santiago de Compostela, Spain). All analyses were performed in triplicate.

### 3.9. Statistical Analysis

Statistical analysis was performed using IBM SPSS Statistics 24.0 for Windows (SPSS Inc., 2016, Chicago, Illinois, USA). Significant differences between the values of all parameters were determined at *p* ≤ 0.05 according to the one-way ANOVA with the posthoc Turkey HSD Test. The results were expressed as mean ± standard deviation.

Data from acid and iodine values, yield, and FA profiles attained from different chromatographic (GC-FID and GC/MS) and proton nuclear magnetic resonance (^1^H-NMR) techniques were subjected to principal component analysis (PCA) using XLSTAT Software (Addinsoft, NY, USA) to examine the differences amongst *Camellia* species (*C. japonica*, *C. sasanqua*, *C. reticulata, and C. hiemalis*).

## 4. Conclusions

In recent years, the commercial interest in high-quality vegetable oils such as those obtained from *Camellia* seeds increased, which is associated with their healthy properties. Economic aspects and beneficial effects of this kind of vegetable oil have provoked interest into the study of them, both by researchers and industry. In this sense, the studies are focused on the characterization of the fatty acid profile and other quality parameters such as acid and iodine values of different species of *Camellia* because they are critical factors involved in oil quality. Four species of *Camellia* grown in the northwest of Spain were studied, with *C. japonica* and *C. sasanqua* being the most abundant, and *C. reticulata* and *C. hiemalis* the least abundant species. In general, results showed quite similar profiles of FAs in the four species, with higher contents of unsaturated FAs (UFAs > 85%), especially highlighting the contents in *C. japonica* species (87–89%), and a low concentration of saturated FAs (10.1–13.6%). Furthermore, the fatty acid profile obtained showed analogous characteristics with other edible *Camellia* oils commercially available in other regions of the world. In addition to the mentioned FAs profile, the extraction yield (16–32%) and the acid (0.4–5.6 mg KOH/g oil) and iodine (70–92 g I_2_/100 g–oil) values of these *Camellia* oils could indicate that this vegetable oil could be used as a high-quality edible oil and be commercially viable, in addition to the preferential use of the species as ornamental plants.

On the other hand, a large number of analytical tests are currently necessary to ensure the quality of oils. The officially recommended chromatographic methods that are used for the identification and the quantification of FAs from *Camellia* oil are tedious, destructive, and time- and resource-consuming. In the present study, the results of a set of analytical techniques (the chromatographic GC-FID and GC-MS and the spectroscopic NMR) were compared for the characterization of FAs. The combination of analytical results from these techniques with multivariate statistics (chemometrics) was an excellent tool to group the *Camellia* oils according to the different species studied. In other words, ^1^H-NMR combined with PCA showed the best grouping of oils by *Camellia* species and discrimination by location, compared to that of traditional chromatographic techniques. This alternative, nondestructive technique is fast, accurate, and simple to perform, avoiding the problems associated with sample handling and pretreatment of alternative conventional techniques. Thus, the combination of methodology based on ^1^H-NMR and PCA could be a suitable tool for quality control of *Camellia* oils and authentication of *Camellia* species used in oil production.

## Figures and Tables

**Table 1:** Origin and quality parameters of *Camellia* seed oils.

Sample	Species	Origin-Code	Harvest	Extraction Yield	Acid Value	Iodine Value
				(*w*/*w*, %)	(mg KOH/g Oil)	(g I_2_/100 g Oil)
1	*C. japonica*	Cuntis	Sep.	26.0	5.61 ± 0.02 jk	79.1 ± 0.5 de
2	*C. japonica*	EFA-826	Sep.	31.9	0.39 ± 0.00 b	82.2 ± 0.0 g
3	*C. japonica*	EFA-942	Sep.	21.6	1.81 ± 0.02 e	82.2 ± 0.2 g
4	*C. japonica*	Quiñones de León/O Castro-876	Aug.	24.0	5.55 ± 0.04 j	83.2 ± 0.1 gh
5	*C. japonica*	Quiñones de León/O Castro-877	Aug.	24.0	5.66 ± 0.00 k	85.6 ± 0.0 i
6	*C. japonica*	Pazo de Lourizán	Sep.	28.4	5.60 ± 0.01 jk	78.7 ± 0.4 cd
7	*C. japonica*	Pazo de Gandarón	Aug.	23.2	4.52 ± 0.04 i	76.5 ± 0.1 b
8	*C. japonica*	Castelo de Soutomaior	Sep.	19.7	5.61 ± 0.00 jk	80.9 ± 0.2 f
9	*C. japonica*	Pazo de Rubianes–Hob Hope	Nov.	16.1	5.62 ± 0.00 jk	79.4 ± 0.1 de
10	*C. japonica*	Pazo de Rubianes–Augusto Leal	Nov.	17.5	5.63 ± 0.00 jk	78.8 ± 0.5 cd
11	*C. japonica*	Pazo de Rubianes–Momoiro–Bokuhan	Nov.	27.3	5.62 ± 0.00 jk	80.1 ± 0.2 ef
12	*C. japonica*	Pazo de Rubianes–Bento de Amorim	Nov.	16.1	5.62 ± 0.02 jk	70.3 ± 0.4 a
13	*C. japonica*	Pazo de Rubianes–Royal Velvet	Nov.	24.1	5.61 ± 0.00 jk	83.1 ± 0.3 gh
14	*C. sasanqua*	EFA-826	Sep.	30.1	0.52 ± 0.00 c	89.8 ± 0.1 j
15	*C. sasanqua*	EFA-942	Sep.	25.0	1.07 ± 0.00 d	82.3 ± 0.0 g
16	*C. sasanqua*	Pazo de A Saleta	Oct.	22.1	2.17 ± 0.01 f	92.0 ± 0.5 k
17	*C. sasanqua*	Pazo de Rubianes	Nov.	26.1	3.41 ± 0.06 g	83.9 ± 0.4 h
18	*C. reticulata*	San Vicente do Mar	Oct.	16.6	3.68 ± 0.01 h	77.2 ± 0.3 b
19	*C. hiemalis*	Pazo de Rubianes	Nov.	22.6	5.64 ± 0.00 jk	83.0 ± 0.4 gh

EFA: Estación Fitopatolóxica Areeiro; results are expressed as mean ± standard deviation (*n* = 3). Different letters (a–k) in same column indicate statistically significant differences between samples (*p* < 0.05).

**Table 2:** FAs composition by GC/FID, expressed as % total fatty acids.

Sample	C14:0	C16:0	C16:1	C18:0	C18:1	C18:2	C18:3	C20:0	C20:1	∑SFA	MUFA	PUFA	∑UFA
1	0.06 ± 0.01 bc	8.24 ± 0.26 a	0.10 ± 0.02 a–c	2.05 ± 0.05 b–d	82.20 ± 0.66 f–h	5.56 ± 0.12 c–e	0.29 ± 0.02 a–c	0.05 ± 0.01 ab	0.29 ± 0.02 ab	10.40	82.59	5.85	88.45
2	0.06 ± 0.01 bc	9.17 ± 0.05 d–f	0.10 ± 0.01 a–c	2.43 ± 0.09 g–i	81.59 ± 0.48 e–h	5.12 ± 0.09 b–d	0.23 ± 0.02 a	0.05 ± 0.01 ab	0.57 ± 0.07 f	11.70	82.26	5.35	87.61
3	0.04 ± 0.01 a	9.46 ± 0.23 e–g	0.12 ± 0.01 a–c	2.36 ± 0.08 f–h	80.96 ± 0.47 c–g	5.65 ± 0.06 e	0.31 ± 0.03 a–d	0.04 ± 0.01 a	0.36 ± 0.03 b–d	11.90	81.44	5.96	87.40
4	0.07 ± 0.01 c	9.80 ± 0.11 gh	0.09 ± 0.01 ab	2.14 ± 0.09 c–f	81.07 ± 0.56 d–g	6.41 ± 0.07 f	0.30 ± 0.04 a–d	0.08 ± 0.01 bc	0.37 ± 0.03 b–d	12.09	81.53	6.71	88.24
5	0.07 ± 0.01 c	9.53 ± 0.08 fg	0.12 ± 0.01 a–c	2.11 ± 0.06 c–e	81.12 ± 0.47 d–g	6.37 ± 0.07 f	0.25 ± 0.03 ab	0.07 ± 0.01 a–c	0.24 ± 0.03 a	11.78	81.48	6.62	88.10
6	0.06 ± 0.01 bc	9.26 ± 0.05 d–f	0.13 ± 0.01 bc	2.29 ± 0.07 e–g	81.06 ± 0.56 d–g	5.61 ± 0.05 de	0.32 ± 0.02 a–d	0.07 ± 0.01 a–c	0.33 ± 0.02 a–c	11.67	81.51	5.93	87.44
7	0.05 ± 0.01 ab	10.41 ± 0.22 ij	0.18 ± 0.02 d	2.28 ± 0.07 defg	78.88 ± 0.33 b–d	7.12 ± 0.09 gh	0.26 ± 0.04 abc	0.05 ± 0.01 ab	0.28 ± 0.02 ab	12.78	79.33	7.38	86.72
8	0.05 ± 0.01 ab	9.05 ± 0.05 c–e	0.10 ± 0.01 a–c	2.46 ± 0.05 g–i	79.18 ± 0.59 b–d	7.43 ± 0.06 h	0.32 ± 0.03 a–d	0.09 ± 0.01 c	0.53 ± 0.03 ef	11.64	79.81	7.75	87.56
9	0.05 ± 0.01 ab	8.21 ± 0.11 a	0.12 ± 0.01 a–c	1.85 ± 0.05 ab	83.04 ± 0.54 gh	5.08 ± 0.08 bc	0.35 ± 0.03 b–e	0.05 ± 0.01 ab	0.44 ± 0.03 c–e	10.15	83.59	5.43	89.02
10	0.05 ± 0.01 ab	9.43 ± 0.11 e–g	0.12 ± 0.01 a–c	2.61 ± 0.04 ij	81.65 ± 0.64 e–h	5.83 ± 0.08 e	0.33 ± 0.02 a–d	0.06 ± 0.01 a–c	0.44 ± 0.04 c–e	12.14	82.20	6.16	88.36
11	0.07 ± 0.01 c	9.13 ± 0.07 def	0.14 ± 0.01 cd	1.72 ± 0.06 a	82.58 ± 0.80 f–h	5.05 ± 0.14 b	0.25 ± 0.04 ab	0.06 ± 0.01 a–c	0.53 ± 0.03 ef	10.98	83.25	5.30	88.55
12	0.06 ± 0.01 bc	8.67 ± 0.08 bc	0.10 ± 0.01 a–c	3.88 ± 0.09 m	83.62 ± 1.26 h	3.91 ± 0.06 a	0.32 ± 0.03 a–d	0.08 ± 0.01 c	0.44 ± 0.04 c–e	12.69	84.16	4.23	88.39
13	0.06 ± 0.01 bc	8.99 ± 0.10 b–d	0.12 ± 0.01 a–c	2.73 ± 0.03 j	82.86 ± 1.16 gh	5.06 ± 0.07 bc	0.28 ± 0.06 a–c	0.06 ± 0.01 a–c	0.44 ± 0.04 c–e	11.83	83.41	5.34	88.76
14	0.05 ± 0.01 abc	8.59 ± 0.16 ab	0.07 ± 0.01 a	2.12 ± 0.07 c–e	80.54 ± 0.46 c–f	6.82 ± 0.12 fg	0.30 ± 0.01 a–d	0.06 ± 0.01 a–c	0.57 ± 0.05 f	10.82	81.18	7.12	88.30
15	0.05 ± 0.01 abc	8.86 ± 0.10 b–d	0.10 ± 0.01 a–c	2.57 ± 0.05 h–j	79.00 ± 0.48 b–d	7.44 ± 0.09 h	0.45 ± 0.04 ef	0.05 ± 0.01 ab	0.82 ± 0.05 g	11.53	79.93	7.89	87.81
16	0.06 ± 0.01 bc	9.05 ± 0.08 c–e	0.13 ± 0.02 bc	2.48 ± 0.07 g–i	78.68 ± 0.53 bc	8.00 ± 0.09 i	0.31 ± 0.03 a–d	0.08 ± 0.01 bc	0.52 ± 0.03 ef	11.66	79.33	8.31	87.64
17	0.07 ± 0.01 c	10.77 ± 0.09 j	0.11 ± 0.02 a–c	1.95 ± 0.12 bc	79.36 ± 1.20 b–e	6.95 ± 0.13 gh	0.36 ± 0.05 c–e	0.06 ± 0.01 abc	0.43 ± 0.03 c–e	12.84	79.90	7.31	87.22
18	0.05 ± 0.01 ab	10.32 ± 0.11 i	0.11 ± 0.01 a–c	3.17 ± 0.07 k	77.97 ± 0.76 b	7.18 ± 0.07 gh	0.41 ± 0.04 d–f	0.04 ± 0.01 a	0.35 ± 0.04 a–c	13.58	78.43	7.59	86.01
19	0.06 ± 0.01 bc	10.20 ± 0.11 hi	0.13 ± 0.01 bc	1.85 ± 0.06 ab	79.23 ± 0.51 b–d	7.12 ± 0.10 gh	0.36 ± 0.01 c–e	0.07 ± 0.01 abc	0.44 ± 0.04 c–e	12.17	79.79	7.49	87.28

∑SFA: total saturated fatty acids. MUFA: monounsaturated fatty acids. PUFA: polyunsaturated fatty acid. ∑UFA: total unsaturated fatty acids. (Results as sums of means); results are expressed as mean ± standard deviation (*n* = 3). Different letters (a–m) in same column indicate statistically significant differences between samples (*p* < 0.05).

**Table 3:** FAs composition by GC/MS, expressed as % total fatty acids.

Scheme 16.	C16:0	C18:0	C18:1 ω-9 *cis*	C18:1 ω-9 *trans*	C18:2 ω-6,-9	C20:1 ω-9	∑SFA	MUFA	PUFA	∑UFA
1	6.69 ± 0.00 c	1.66 ± 0.02 ef	87.1 ± 0.1 gh	0.72 ± 0.03 d–f	3.59 ± 0.02 d	0.25 ± 0.01 ab	8.35	88.07	3.59	91.65
2	7.44 ± 0.07 fg	1.94 ± 0.04 ij	86.5 ± 0.1 fg	0.76 ± 0.04 d–g	3.08 ± 0.03 bc	0.24 ± 0.02 bc	9.38	87.53	3.08	90.62
3	6.84 ± 0.03 cd	1.64 ± 0.02 d–f	87.7 ± 0.1 hi	0.66 ± 0.03 c–e	3.12 ± 0.05 bc	ND	8.48	88.43	3.12	91.52
4	7.80 ± 0.06 hi	1.68 ± 0.01 e–g	85.3 ± 0.2 c–e	0.92 ± 0.06 h	4.06 ± 0.09 ef	0.22 ± 0.00 a	9.48	86.43	4.06	90.52
5	7.51 ± 0.03 gh	1.58 ± 0.01 de	86.1 ± 0.1 ef	0.90 ± 0.07 gh	3.75 ± 0.04 de	0.20 ± 0.01 a	9.09	87.17	3.75	90.91
6	8.04 ± 0.17 i	1.49 ± 0.02 cd	85.5 ± 0.3 de	0.74 ± 0.01 d–f	4.27 ± 0.09 f–h	ND	9.53	86.20	4.27	90.47
7	8.46 ± 0.01 j	0.89 ± 0.02 a	84.6 ± 0.1 c	0.89 ± 0.02 gh	4.35 ± 0.06 f–h	ND	9.35	85.53	4.35	89.86
8	6.92 ± 0.19 c–e	1.86 ± 0.03 g–i	87.6 ± 0.4 hi	0.66 ± 0.03 c–e	3.01 ± 0.14 b	ND	8.78	88.23	3.01	91.22
9	6.07 ± 0.02 a	1.27 ± 0.01 b	89.2 ± 0.1 j	0.62 ± 0.04 c–e	2.79 ± 0.06 b	ND	7.34	89.87	2.79	92.66
10	7.45 ± 0.11 fg	2.05 ± 0.08 j	85.5 ± 0.6 de	1.18 ± 0.06 i	3.49 ± 0.39 cd	0.29 ± 0.01 c	9.50	87.03	3.49	90.50
11	7.17 ± 0.08 ef	1.39 ± 0.05 bc	87.4 ± 0.3 hi	0.80 ± 0.06 f–h	2.92 ± 0.11 b	0.34 ± 0.01 c	8.56	88.50	2.92	91.44
12	6.38 ± 0.05 b	2.76 ± 0.05 l	87.9 ± 0.2 i	0.92 ± 0.01 gh	2.08 ± 0.09 a	ND	9.14	88.77	2.08	90.86
13	7.06 ± 0.11 de	2.02 ± 0.03 j	87.1 ± 0.3 g–i	0.77 ± 0.02 e–h	3.02 ± 0.14 b	ND	9.08	87.90	3.02	90.92
14	7.11 ± 0.10 de	1.78 ± 0.03 f–h	85.6 ± 0.2 de	0.53 ± 0.01 a–c	4.54 ± 0.05 gh	0.41 ± 0.00 d	8.89	86.57	4.54	91.11
15	7.17 ± 0.03 e	1.97 ± 0.01 j	85.2 ± 0.1 cd	0.57 ± 0.00 b–d	4.70 ± 0.03 h	0.38 ± 0.00 d	9.14	86.13	4.70	90.86
16	7.50 ± 0.11 gh	1.84 ± 0.04 g–i	83.4 ± 0.3 b	0.42 ± 0.04 a	6.53 ± 0.15 i	0.33 ± 0.01 c	9.34	84.13	6.53	90.66
17	8.39 ± 0.06 j	1.46 ± 0.05 bc	85.2 ± 0.3 cd	0.75 ± 0.05 d–h	4.21 ± 0.20 fg	ND	9.85	85.93	4.21	90.15
18	9.32 ± 0.07 k	2.64 ± 0.02 kl	83.3 ± 0.2 b	0.46 ± 0.02 ab	4.03 ± 0.15 ef	0.21 ± 0.01 a	11.96	84.00	4.03	88.04
19	7.84 ± 0.07 i	1.28 ± 0.03 b	86.0 ± 0.1 ef	0.65 ± 0.04 c–f	4.23 ± 0.03 fg	ND	9.12	86.67	4.23	90.88

∑SFA: total saturated fatty acids. MUFA: monounsaturated fatty acids. PUFA: polyunsaturated fatty acid. ∑UFA: total unsaturated fatty acids. Results as sums of means; results are expressed as mean ± standard deviation (*n* = 3). ND: not detected. Different letters (a–k) in same column indicate statistically significant differences between samples (*p* < 0.05).

**Table 4:** FAs composition by ^1^H-NMR, expressed as % total fatty acids.

Sample	Species	C18:1 (MUFA)	C18:2	C18:3	∑SFA	PUFA	∑UFA
1	*C. japonica*	89.9 ± 0.4 f	5.78 ± 0.19 b–e	ND	12.36	5.78	95.63
2	*C. japonica*	86.3 ± 0.4 e	4.33 ± 0.00 a	ND	12.92	4.33	90.63
3	*C. japonica*	86.0 ± 0.2 de	5.33 ± 0.00 bc	ND	12.64	5.33	91.35
4	*C. japonica*	94.3 ± 0.3 g	7.33 ± 0.00 hi	ND	15.25	7.33	101.63
5	*C. japonica*	96.4 ± 0.6 hi	7.33 ± 0.00 hi	ND	14.75	7.33	103.69
6	*C. japonica*	86.4 ± 0.2 e	7.11 ± 0.19 g–i	ND	13.75	7.11	93.46
7	*C. japonica*	85.6 ± 0.9 de	6.67 ± 0.33 e–h	ND	14.25	6.67	92.30
8	*C. japonica*	89.6 ± 0.3 f	5.33 ± 0.00 bc	ND	13.36	5.33	94.96
9	*C. japonica*	90.4 ± 0.7 f	5.22 ± 0.19 b	ND	11.64	5.22	95.58
10	*C. japonica*	98.1 ± 0.8 i	6.33 ± 0.33 d–g	ND	14.69	6.33	104.41
11	*C. japonica*	97.8 ± 0.3 i	5.11 ± 0.19 ab	ND	13.63	5.11	102.91
12	*C. japonica*	94.5 ± 1.0 gh	5.11 ± 0.19 ab	ND	16.02	5.11	99.63
13	*C. japonica*	93.3 ± 0.4 g	5.56 ± 0.19 b–d	ND	13.91	5.56	98.85
14	*C. sasanqua*	84.7 ± 0.1 c–e	6.67 ± 0.00 f–h	ND	12.25	6.67	91.41
15	*C. sasanqua*	83.6 ± 0.1 c	7.67 ± 0.00 i	ND	12.86	7.67	91.24
16	*C. sasanqua*	85.7 ± 0.2 de	10.33 ± 0.00 j	ND	13.80	10.3	96.08
17	*C. sasanqua*	84.1 ± 0.3 cd	7.33 ± 0.00 hi	ND	14.36	7.33	91.41
18	*C. reticulata*	81.0 ± 0.5 b	7.11 ± 0.19 g–i	ND	17.25	7.11	88.07
19	*C. hiemalis*	91.1 ± 1.7 f	7.89 ± 0.77 i	ND	14.58	7.89	98.96
20 *	*C. japonica*	80.7	6.65	0.29	12.4	6.94	87.64
21 **	*C. sasanqua*	82.3	6.20	0.30	11.2	6.50	88.80
22 **	*C. reticulata*	84.5	5.69	0.26	9.58	5.95	90.42
23 **	*C. oleifera*	83.8	7.78	0.41	8.04	8.19	91.96

∑SFA: total saturated fatty acids. MUFA: monounsaturated fatty acids. PUFA: polyunsaturated fatty acid. ∑UFA: total unsaturated fatty acids. Results as sums of means; results are expressed as mean ± standard deviation (*n* = 3). ND: not detected. Different letters (a–j) in same column indicate statistically significant differences between samples (*p* < 0.05). Values from bibliography. Reference: * [3]. ** [32].

**Table 5:** Chemical shift assignment of ^1^H-NMR for FAs.

Peak	δ (ppm)	Multiplicity	Functional Group	Compound
1	5.32	m	–CH=CH–	acyl group
2	5.25	m	–CH–O–COR	glyceryl group
3	4.27	dd	–CH_2_–O–COR	glyceryl group
4	2.74	t	=CH–CH_2_–CH=	acyl group (linoleic and linolenic group)
5	2.29	dt	–OCO–CH_2_–	acyl group
6	2.01	m	–CH_2_–CH=CH–	acyl group
7	1.61	m	–OCO–CH_2_–CH_2_–	acyl group
8	1.29	m	–(CH_2_)n–	acyl group
9	0.98	t	–CH=CH–CH_2_–CH_3_	linoleic acyl group
9	0.88	t	–CH_2_–CH_2_–CH_2_–CH_3_	saturated oleic except linoleic acyl group

d: doublet; t: triplet; m: multiplet; dt: double of triplet; dd: doublet of doublet.

**Table 6:** Signal identification and quantification according to Barison’s method.

Fatty Acid	Label	^1^H NMR Signal	Reference Area (Signal)	Subtration
Linolenic	E	0.98 ppm	22.2	--
Linoleic	A	2.74 ppm	33.3	2 × linoleic
Oleic	C	2.01 ppm	16.7	linolenic and linoleic
Saturated	B	2.29 ppm	33.3	linolenic + linoleic + oleic

## Data Availability

Data is contained within the article.
